# Expert and Novice Performers Respond Differently to Attentional Focus Cues for Speed Jump Roping

**DOI:** 10.3389/fpsyg.2018.02370

**Published:** 2018-11-29

**Authors:** Kaylee F. Couvillion, Jeffrey T. Fairbrother

**Affiliations:** Motor Behavior Laboratory, Department of Kinesiology, Recreation, and Sport Studies, University of Tennessee, Knoxville, Knoxville, TN, United States

**Keywords:** attentional focus, focus cues, motor performance, motor skills, expert performance, jump rope, sport, external focus

## Abstract

Directing attention to an external focus has been shown to facilitate motor performance. For expert performers, however, results have been mixed. Additionally, little is currently known about how focus cues affect the performance of complex continuous whole-body coordination tasks involving object manipulation such as jump roping. The purpose of this study was to examine the effects of attentional focus cues on single-rope speed jumping by experts and novices. The cues directed attention toward the upper (UP) or lower (LB) body and either internally (IN) or externally (EX). Participants (*N* = 30) completed bouts of speed jumping during a baseline trial and under experimental conditions (UPIN, UPEX, LBIN, LBEX). Jumps and errors were recorded for each trial. Number of jumps (NJ) and errors (NE) were analyzed using separate Friedman’s Tests comparing for each group to compare trials, with Wilcoxon Signed-Rank Tests for *post hoc* comparisons. Cumulative number of jumps (CNJ) and errors (CNE) for each condition were compared using separate Friedman’s Tests with Wilcoxon Signed-Rank Tests for *post hoc* comparisons. For experts, baseline NJ was significantly higher than NJ for each trial under the UPIN, UPEX, and LBIN conditions. No differences between baseline NE and any trials were detected. Additionally, no differences were detected between conditions for NJ or NE. For novices, baseline NJ and NE were significantly higher and lower, respectively, compared to Trial 1 under the LBEX condition. Both the UPIN and UPEX conditions produced higher CNJ and lower CNE than the LBIN and LBEX conditions, respectively. Results showed that experts and novices responded in distinctly different patterns to the four conditions. Experts showed degraded performance under the UPIN, UPEX, and LBIN conditions whereas novices only showed temporarily degraded performance under the LBEX condition. These findings may reflect differences in mastery of whole-body coordination and are partially consistent with the *Constrained Action Hypothesis* (CAH) despite not supporting specific predictions related to the benefit of external focus cues.

## Introduction

Coaches, therapists, and trainers regularly use verbal cues to direct a performer’s attention to certain aspects of skill execution. A substantial body of literature has examined the effects of such attention-directing cues on motor performance. Most commonly, attentional focus cues have been categorized by whether they direct attention internally or externally. This distinction has come tobe operationalized as directing attention either toward the control of the movement (internally) or toward the effects of the movement (externally) (Wulf and Prinz, 2001). The majority of experimental results examining the effects of these two different attentional foci have revealed that an external focus facilitates performance compared to an internal focus (for a review see Wulf, 2013). The external focus benefit has been documented for a variety of discrete skills such as dart throwing, golf putting, free throw shooting, and standing long jump (e.g., Zachry et al., 2005; Poolton et al., 2006; Marchant et al., 2007; Becker et al., 2018). A smaller body of research has also shown benefits of an external focus of attention for the performance of continuous tasks such as swimming, jump roping, and balance (Freudenheim et al., 2010; Porter et al., 2016; Rhea et al., in press).

One prominent explanation for attentional focus effects has been termed the *Constrained Action Hypothesis* (CAH) (Wulf et al., 2001), which argued that an external focus facilitates performance by allowing the motor system to take advantage of automated processes related to movement control. The CAH also argued that an internal focus prompts performers to consciously control movements, which disrupts processes that would be more optimally controlled in an automated fashion. The *conscious processing explanation* (Poolton et al., 2006) has also been forwarded as a possible explanation of observed external focus benefits. According to this viewpoint, external focus instructions confer performance advantages over internal focus instructions because they have typically been shorter and have thus imposed a lower working memory load.

When the effects of attentional focus instructions have been examined using expert performers, results have been mixed. Some studies have reported findings consistent with an external focus benefit (Wulf and Su, 2007; Halperin et al., 2017), while others have revealed no differences in performance between conditions or, occasionally, superior performance under control conditions compared to both internal and external focus conditions (Wulf, 2008; Porter and Sims, 2013; Winkelman et al., 2017). Moreover, research examining the attentional focus behaviors adopted by expert performers during practice and competition has revealed that the use of internal foci is common (Bernier et al., 2011, 2016; Fairbrother et al., 2016; Guss-West and Wulf, 2016). Existing evidence indicates that experts can adopt complex attentional strategies that extend beyond a simple internal vs. external dichotomy. One area of research that has begun to explore more complex manipulations of attentional focus has examined how the proximity of an external focus cue influences performance. More distal focus cues are those that direct attention farther away from the performer’s body, whereas proximal cues direct attention closer to the body. Typically, external focus benefits have been found to be more pronounced for more distal focus targets. For example, McNevin et al. (2003) reported superior balance performance by a group instructed to focus on markers placed farther away from their feet compared to a group instructed to focus on markers placed closer to their feet. Similarly, Porter et al. (2012) found that standing long jump performance was enhanced by a cue to focus on a distal target (3 m) compared to a cue to focus on jumping away from the starting line.

When proximity of focus has been applied to internal focus cues, results have been mixed. Pelleck and Passmore (2017) found that golf putting performance was more accurate for novices using a proximal internal focus compared to a distal internal focus or an external focus for the longer of two putts. In contrast, no differences were seen for experts. These results combined with the mixed results from studies on expert performers (Wulf and Su, 2007; Wulf, 2008; Porter and Sims, 2013; Halperin et al., 2017; Winkelman et al., 2017) indicate that attentional focus effects may not generalize to highly skilled populations. Presumably, expert performers have developed attentional focus strategies that support their high levels of skilled performance. Although it is possible, as some have argued (Guss-West and Wulf, 2016), that their performance would be further enhanced through the systematic adoption of an external focus of attention, the existing literature showing a lack of benefit for experts suggests that such an approach is not yet warranted. Another possibility is that the majority of previous research on attentional focus effects has been overly reductive. The internal vs. external dichotomy does not appear to be nuanced enough to systematically address attentional focus effects in experts.

One approach to address this gap is to identify a set of attentional focus cues consistent with the attentional focus target areas used by experts that also lend themselves to experimental research. Competitive speed jump roping offers such an opportunity because athletes commonly focus their attention on their hands or wrists, the jump rope handles, their feet, and the sounds of foot contact with the ground. These attentional target areas^1^ provide a straightforward way to compare the effects of multiple task-relevant internal and external focus cues. The purpose of the present experiment was, therefore, to examine the effects of upper- and lower-body internal and external focus cues on motor performance by highly skilled experts. A novice group was included to illustrate potential ways in which responses to cues might differ for different skill levels. Based on the previous research on attentional focus effects in experts, it was expected that none of the cues would improve performance compared to baseline. In contrast, the majority of attentional focus research involving novices has shown that an external focus cue facilitates performance. It was therefore expected that both external cues would improve performance compared to baseline for the novices. Consistent with Pelleck and Passmore (2017) findings, it was also expected that novices would show degraded performance compared to baseline when using the lower-body internal cue. Previous research showing advantages of more distal *external* cues led to the expectation that the lower-body external focus cue (foot sounds) would facilitate performance compared to the upper-body external focus cue (handles) for the novices. Presumably, the inclusion of both upper- and lower-body external foci compared to their internal counterparts increased the opportunity to determine if attentional focus effects in experts are perhaps influenced by the location of the focus cue.

## Materials and Methods

### Participants

Participants were 30 expert (*n* = 15) and novice (*n* = 15) jump ropers between the ages of 18 and 30 who gave voluntary informed consent upon enrolling in the study. The expert group was comprised of jump rope athletes who had competed at national and/or international levels in single rope speed jumping. The novice group was comprised of students from a university in the southeastern US who had not previously attempted single rope speed jumping. The protocol and informed consent form were approved by the University of Tennessee, Knoxville Institutional Review Board.

### Task

The experimental task required participants to single-rope speed jump for a duration of 15 s. Single-rope speed jumping involves jumping so that the rope passes underneath the feet while the competitor performs and alternating step pattern (World Jump Rope Federation [WJRF] 2017a). The goal of the task was to complete as many steps as possible during the 15 s bout.

### Procedure

Upon arrival at the testing site (e.g., an indoor gymnasium), participants provided voluntary informed consent and were then given a description of study procedures. Participants were told that they would be asked to direct their attention to different targets while completing the task under the experimental conditions. They were instructed that their goal was to complete as many jumps as possible during each 15 s trial. Participants were also told to continue the trial despite making any mistakes. Each participant was tested independently. Prior to beginning the trials, the participant completed a warm up consisting of self-paced speed jumping for durations of 60, 45, 30, and 15 s. They were also allowed to perform additional warm up jumping or stretching if they desired.

Each participant completed a total of nine 15 s trials, separated by 3 min rest periods. Trials were video-recorded using a first generation iPad (Apple; Cupertino, CA, United States). The first trial for each participant served as a baseline^2^. Following this trial, participants completed two trials in each of the remaining four attentional focus conditions. Each of the attentional focus conditions were completed once in a counterbalanced order using a Latin square design. Following completion of one trial in each attentional focus condition, the conditions were repeated a second time in the same order. For example, if a certain condition was completed on the second trial (directly following the baseline trial), that same condition was repeated on the sixth trial, following the other three focus conditions^3^.

Just prior to each trial, participants were given the appropriate attentional focus instruction for the condition. Instructions for the baseline trial were to “simply perform as many jumps as you can during the 15 s bout.” Instructions given prior to the two upper-body focus conditions were similar to those used by Porter et al. (2016). The instruction for the upper-body external focus condition (UPEX) was to “focus on making small, fast ovals with the tips of your handles.” Instruction for the upper-body internal focus condition (UPIN) was to “focus on making small, fast ovals with your wrists.” For novices, the UPIN and UPEX instructions were simplified to ensure proper understanding. Specifically, the UPIN instruction for novices was to “focus on making fast rotations with your wrists,” and the UPEX instruction was to “focus on making fast rotations with the tips of your handles”^4^. Instructions for the lower-body external focus condition (LBEX) were to “focus on creating a fast sound with your shoes on the floor”. Instructions for the lower-body internal focus condition (LBIN) were to “focus on making fast movements with your feet.”

Prior to each trial, the official World Jump Rope Federation (WJRF) 1 × 180 single-rope speed timing track was played. The “go” signal was denoted by a loud beep, and the “stop” signal was denoted by the word “fifteen” (indicating that 15 s had passed). Performance was monitored according to the scoring protocol outlined in the World Jump Rope Federation [WJRF] (2017b). During each trial, the experimenter counted the number of successful jumps by recording right foot contacts using a manual tally counter (H-102 Professional Model Japanese Talley Counter). Errors were also recorded. Each trial was video-recorded to confirm jump and error counts. Participants were not given feedback about their performances.

Following completion of the nine trials, participants completed a questionnaire, indicating whether or not they were able to comply with each instructed focus cue. To account for the possibility that some participants might interpret adherence as equivalent to *absolute* adherence under each condition, they were also asked to report the percentage of time they were successful in maintaining each focus. The questionnaire also asked participants to indicate which conditions they found to be most and least helpful, their preferred focus, and the condition(s) with which they were familiar.

### Data Treatment and Analysis

The primary dependent measures were the number of jumps (NJ) and number of errors (NE) for each trial. NJ and NE were analyzed using separate Friedman’s Tests for each group to examine differences between all trials (nine total trials). Following significant results of the Friedman’s omnibus test, *post hoc* procedures were completed using separate Wilcoxon Signed-Rank Tests comparing baseline scores to each trial within each condition. The goal of these comparisons was to determine which, if any, trials within each experimental condition significantly changed performance compared to baseline performance tested at the outset of the study. Jumps and errors were also summed across the two trials for each condition to calculate the cumulative number of jumps (CNJ) and errors (CNE). CNJ and CNE under each condition were analyzed using separate Friedman’s Tests. Following significant findings, *post hoc* procedures were completed using separate Wilcoxon Signed-Rank Tests for specific comparisons between internal and external cues associated with the upper-body and lower-body locations (UPIN vs. UPEX and LBIN vs. LBEX) and between upper- and lower-body cues associated with the internal and external directions (UPIN vs. LBIN and UPEX vs. LBEX). The alpha level was set to 0.05 for all analyses. Responses from the questionnaire were tabulated and presented descriptively.

## Results^5^

### Number of Jumps

**FIGURE 1:**
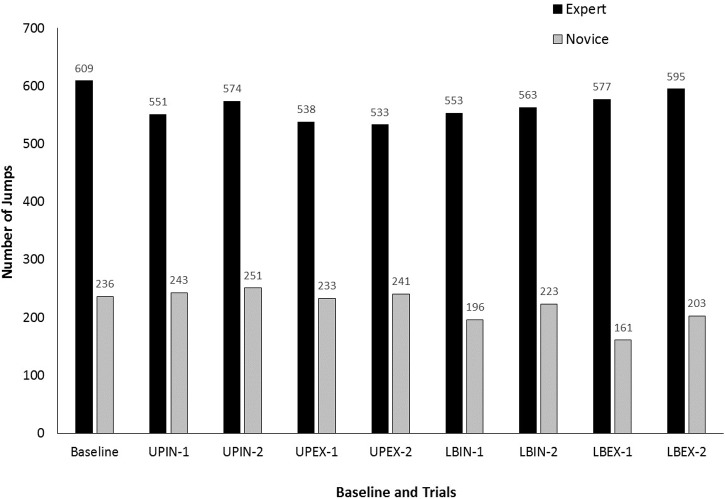
Number of jumps (NJ) for each group during baseline and each trial of each attentional focus condition (UPIN, upper body, internal; UPEX, upper body, external; LBIN, lower body, internal; LBEX, lower body, external). The “-1” and “-2” labels indicate the first and second trials, respectively, for each condition.

Figure 1 shows the number of jumps (NJ) completed during baseline and each trial of each attentional focus condition. For the experts, all of the attentional focus conditions produced lower NJ compared to baseline. These observations were supported by a significant difference in NJ depending upon trial, χ^2^(8) = 24.77, *p* = 0.002. *Post hoc* comparisons revealed significant differences between baseline and UPIN-1 (*Z* = -2.91, *p* = 0.004), UPIN-2 (*Z* = -2.05, *p* = 0.041), UPEX-1 (*Z* = -3.08, *p* = 0.002), UPEX-2 (*Z* = -2.91, *p* = 0.004), LBIN-1 (*Z* = -2.56, *p* = 0.011), and LBIN-2 (*Z* = -2.56, *p* = 0.011). No significant differences were detected between baseline and LBEX-1 (*p* = 0.058) and LBEX-2 (*p* = 0.128). For the novices, the upper-body cues produced similar NJ compared to baseline while the lower-body cues produced lower NJ. These observations were supported by a significant difference in NJ depending upon trial, χ^2^(8) = 34.88, *p* < 0.001. *Post hoc* comparisons revealed significant differences between baseline and LBEX-1 (*Z* = -3.18, *p* = 0.001). None of the other trials differed significantly from baseline (*p*-values from 0.086 to 0.925).

**FIGURE 2:**
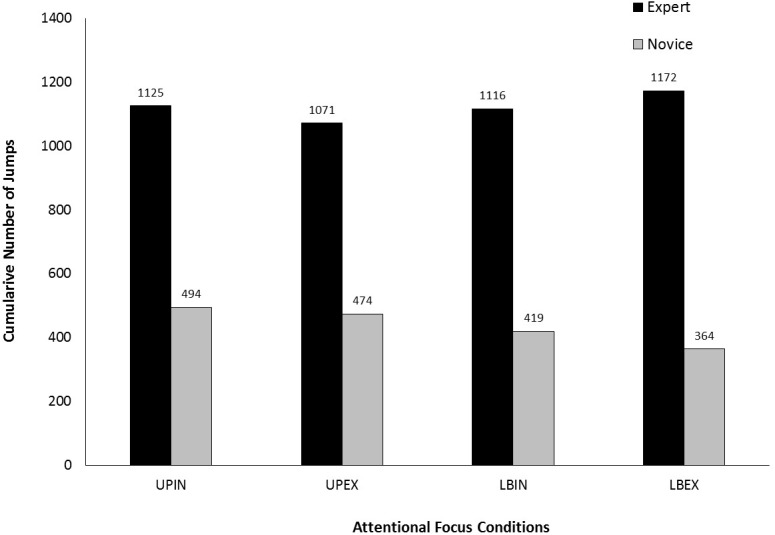
Cumulative number of jumps (CNJ) for both trials under each attentional focus condition (UPIN, upper body, internal; UPEX, upper body, external; LBIN, lower body, internal; LBEX, lower body, external).

Figure 2 shows cumulative number of jumps (CNJ) for each of the four attentional focus conditions. For the experts, CNJ were similar under each condition. These observations were supported by no significant difference in CNJ dependent upon condition, χ^2^(3) = 5.11, *p* = 0.164. For the novices, CNJ was highest for the UPIN condition and lowest for the LBEX condition. CNJ was higher for the upper-body conditions, which were similar to one another, compared to the lower-body conditions. CNJ was also higher for the LBIN condition compared to the LBEX condition. These observations were supported by a significant difference in CNJ depending upon condition, χ^2^(3) = 16.47, *p* = 0.001. *Post hoc* comparisons revealed significant differences between the lower- and upper-body internal conditions (*Z* = -2.61, *p* = 0.009) and between the lower- and upper-body external conditions (*Z* = -2.70, *p* = 0.007). No significant differences were detected between the two upper-body conditions (*p* = 0.551) or between the two lower-body conditions (*p* = 0.053).

### Number of Errors

**FIGURE 3:**
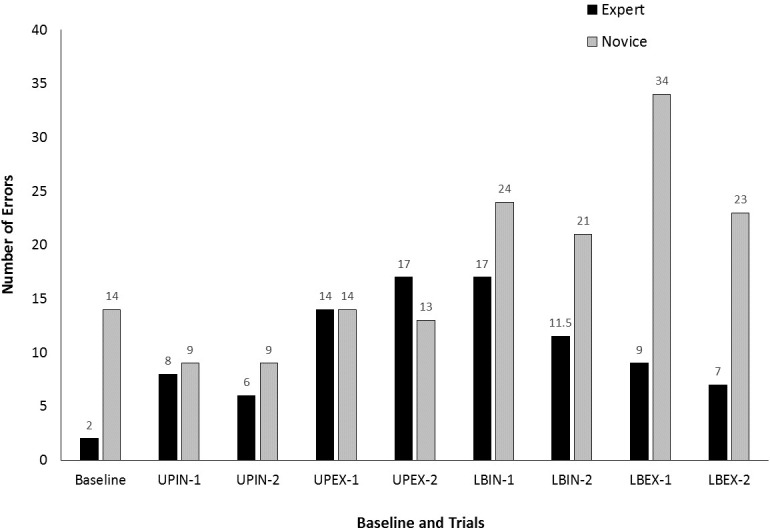
Number of errors (NE) for each group during baseline and each trial of each attentional focus condition (UPIN, upper body, internal; UPEX, upper body, external; LBIN, lower body, internal; LBEX, lower body, external). The “-1” and “-2” labels indicate the first and second trials, respectively, for each condition.

Figure 3 shows the number of errors (NE) completed during baseline and each trial of each attentional focus condition. For the experts, all of the attentional focus conditions produced higher NE compared to baseline. These differences did not, however, result in a significant difference in NE, χ^2^(8) = 14.22, *p* = 0.76. For the novices, the upper-body conditions produced smaller or similar NE compared to baseline while the lower-body conditions produced higher NE. These observations were supported by a significant difference in NE depending upon trial, χ^2^(8) = 35.34, *p* < 0.001. *Post hoc* comparisons revealed significant differences between baseline and the first trial under the LBEX condition (*Z* = -3.13, *p* = 0.002). None of the other trials differed significantly from baseline (*p*-values from 0.136 to 0.963).

**FIGURE 4:**
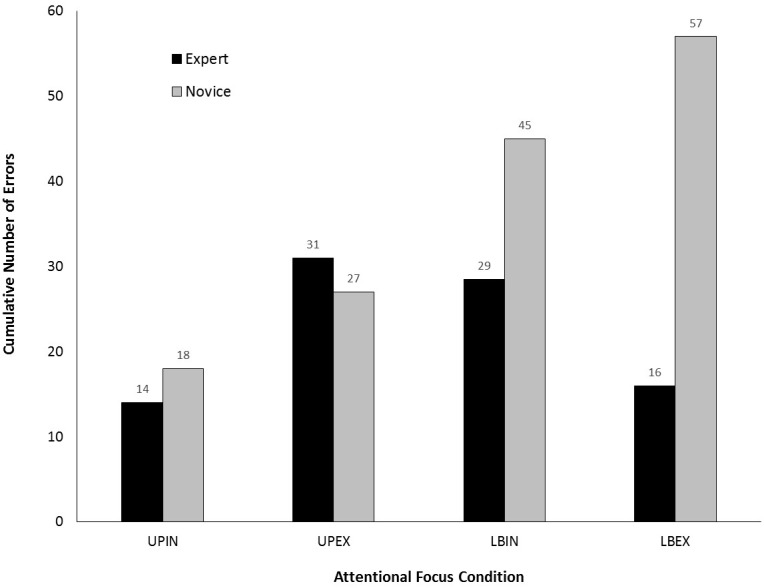
Cumulative number of errors (CNE) for both trials under each attentional focus condition (UPIN, upper body, internal; UPEX, upper body, external; LBIN, lower body, internal; LBEX, lower body, external).

Figure 4 shows cumulative number of errors (CNE) for both trials of each of the four attentional focus conditions. For the experts, CNE were similar under each condition. These observations were supported by the lack of significant difference in CNE, χ^2^(3) = 3.49, *p* = 0.322. For the novices, CNE was lowest for the UPIN condition and highest for the LBEX condition. CNE was lower for the upper-body conditions compared to the lower-body conditions. CNE was also lower for the two internal conditions compared to the respective external conditions. These observations were supported by a significant difference in CNE dependent upon condition, χ^2^(3) = 22.07, *p* < 0.001. *Post hoc* comparisons revealed significant differences between the lower- and upper-body internal conditions (*Z* = -2.61, *p* = 0.009) and between the lower- and upper-body external conditions (*Z* = -3.03, *p* = 0.002). No significant differences were detected between the two upper-body conditions (*p* = 0.163) or between the two lower-body conditions (*p* = 0.102).

### Perceived Success in Using Attentional Focus Cues

Table 1 shows the number of participants who reported that they were able to focus on the instructed attentional target. Perceived adherence in the expert group ranged from 80% of participants in the UPEX condition to 100% in the LBEX condition. Perceived adherence in the novice group was 80% in both lower-body conditions and 100% in both upper-body conditions. Participants were also asked to report the percentage of time they were successful in using the instructed cue during each condition. Of the participants who reported “No” to the perceived adherence question for a condition, all but one expert and two novices reported that they were actually successful 26–75% of the time in that condition. Table 2 shows the number of participants in each percentage range for the question related to success in using the instructed cue. The number of expert participants who reported success in using the instructed cue at least 50% of the time ranged from 11 (UPEX) to 14 (LBEX) which corresponded to 73–93% of responses. The number of novice participants who reported success in using the instructed cue at least 50% of the time ranged from 11 (LBIN and LBEX) to 15 (UPIN) which corresponded to 73–100% of responses.

**Table 1:** Number of participants reporting “yes” or “no” to the question about whether or not they were able to focus on the instructed attentional target.

		Perceived adherence			
		UPIN	UPEX	LBIN	LBEX
Experts	Yes	14	12	13	15
	No	1	3	2	0
Novices	Yes	15	15	12	12
	No	0	0	3	3


**Table 2:** Number of participants in each range for responses to the question about the percent of time they were successful using the cue in each attentional focus condition.

		Success using cue			
		UPIN	UPEX	LBIN	LBEX
Experts	0–25%	0	0	1	0
	26–50%	2	4	1	1
	51–75%	5	4	3	3
	76–100%	8	7	10	11
Novices	0–25%	0	0	2	4
	26–50%	0	1	2	0
	51–75%	4	4	5	8
	76–100%	11	9	6	3


### Familiarity, Perceived Helpfulness, and Preferences Related to Focus Cues

For the experts, 87% reported that they were familiar with at least one of the focus targets. The largest number of experts reported familiarity with the UPIN and LBIN targets and the smallest number reported familiarity with the UPEX and LBEX targets. None of the experts reported familiarity with all four focus targets. For the novices, 87% reported that they were *not* familiar with any of the attentional focus instructions. One novice participant indicated familiarity with the UPIN focus target, and one indicated familiarity with the UPEX and LBIN targets.

Table 3 shows the number of participants who reported each condition as most helpful, least helpful, and preferred. The majority of expert participants indicated that they preferred the LBEX condition (foot sounds) or a combination of multiple foci. Of the four who stated a preference for a combination, all included the UPIN condition in the combination. The LBEX condition was reported to be the most helpful. Of the seven experts who reported LBEX as most helpful, five also indicated it was their preferred condition, one reported a preference for a combination of the LBEX and UPIN conditions, with an emphasis on the former, and one reported a preference for no instruction. Only one expert participant indicated the baseline instruction as the most helpful, which suggests that these participants were perhaps unaware that performance was degraded by several of the attentional focus conditions. The second most helpful condition was the UPIN condition, which was also one of the two for which the most experts reported familiarity. The other condition reported by the most experts as familiar (LBIN) was reported as least helpful by more participants compared to the other conditions (although the UPEX condition was close). There were no systematic relationships between experts’ selections of most helpful, least helpful, and preferred conditions. Nor were there any systematic relationships with familiarity. Novices’ responses, however, revealed clear relationships between helpfulness and preference. All participants’ selections for the most helpful instruction matched their selections for the preferred instruction. The most helpful conditions for novices were the UPIN and UPEX conditions, whereas the LBEX condition was the least helpful. Two participants indicated a preference for the instruction provided during the baseline trial.

**Table 3:** Number of participants who reported each condition as most helpful, least helpful, and preferred.

		Most helpful	Least helpful	Preferred
Experts	Baseline	1	2	2
	UPIN	5	1	2
	UPEX	3	5	1
	LBIN	4	6	2
	LBEX	7	3	5
	Combination	0	0	4
Novices	Baseline	2	0	2
	UPIN	6	1	6
	UPEX	5	3	5
	LBIN	2	2	2
	LBEX	0	9	0
	Combination	0	0	0


## Discussion

Previous research has indicated that an external focus of attention is beneficial for the performance of various motor tasks (for a review see Wulf, 2013). However, tests of attentional focus instructions in performers with high levels of expertise have yielded mixed results (Porter and Sims, 2013; Halperin et al., 2017). The purpose of the current study was to examine the effects of upper-body and lower-body internal and external focus cues on single-rope speed jump rope performance of expert and novice participants. The most important contribution of the current study was a pattern of findings that was consistent with previous literature showing that expert and novice performers responded in distinctly different ways to the various attentional focus cues (Pelleck and Passmore, 2017; Winkelman et al., 2017). Consistent with previous studies involving experts, the present results demonstrated that focus cues either had no effect or degraded performance for experts compared to baseline (e.g., Wulf, 2008; Porter and Sims, 2013). For the experts, decreased number of jumps were seen in both upper-body conditions and the lower-body internal condition. The lower-body external condition did not affect performance. In contrast, novice performance was temporarily degraded (decreased jumps and increased errors) only by the lower-body external condition. The other three conditions produced no effects. The expectation that experts would show no changes from baseline was not supported in three of the conditions. Results did not illustrate a clear pattern with respect to body position since the performance decrements were seen for both upper-body and one lower-body conditions. The expectation that the two external focus cues would facilitate performance compared to baseline for the novices was also not supported. Indeed, the LBEX condition caused a temporary performance decrement seen in both measures. The expectation that the LBIN condition would degrade performance for novices was not supported nor was the expectation that the LBEX condition would facilitate performance compared to the UPEX condition. Results related to the latter expectation actually showed the opposite, with the UPEX condition conferring a benefit. Additionally, the upper-body benefit extended to both the internal and external conditions.

For experts, focusing on the jump rope handles (UPEX), the wrists (UPIN), and the feet (LBIN) all decreased the number of jumps completed. At least two issues may have contributed to the degraded performance. First, only two experts indicated that they were familiar with the specific UPEX instruction. Reduced cue familiarity has previously been shown to degrade performance (Maurer and Munzert, 2013). The instructions to focus on the hands or on the feet were familiar to a much larger number of participants (eight for each condition). Although the number of experts familiar with each cue cannot explain why three cues degraded performance and one did not, it is important to recognize that there were no significant differences between the conditions for experts. Additionally, the observed differences in effects compared to baseline involved three similar effects and one null finding. Thus, it seems reasonable to conclude that the relatively low number of experts familiar with each cue (13–53%) had at least some impact on the experts’ performances. Although the cues were consistent with commonly used target areas used in competitive speed jump rope and jump rope instruction, the specific cues were novel for a number of the participants. It is perhaps unreasonable to expect immediately observable effects given the short duration of typical experimental protocols. Presumably, experts have developed effective attentional strategies over years or even decades of practice and so it follows logically that imposing a novel cue might disrupt immediate performance. Future research should be devoted to assessing the effects of external focus cues after allowing experts ample time to acclimate to using them. At the same time, the current results and previous studies showing no external focus benefits for experts serve to temper advocacy for the widespread adoption of external cues as a quick way to enhance performance.

Second, the instructions for the UPIN, UPEX, and LBIN conditions may have prompted a conscious control strategy. The UPIN and LBIN conditions both directed attention toward control of a specific part of the body. The UPEX condition directed attention toward controlling the rope. In all three cases, it is possible that these instructions disrupted a well-established and automated pattern of whole-body coordination. Both of these possibilities – lack of cue familiarity and a focus on controlling one aspect of performance – are consistent with the types of strategies seen in early stages of learning (Fitts and Posner, 1967) and are generally associated with inferior performance. Another possible contributing factor is that all three cues presumably focus attention on movement technique rather than movement outcome. From this perspective, the cue to focus on the handles may have disrupted performance in a manner similar to what was proposed for internal foci in the Constrained Action Hypothesis (Wulf et al., 2001) despite the fact that it actually promoted an external focus. Accordingly, the distinction between control and outcome foci may be more important for speed jump roping than the distinction between internal and external foci. Thus, the findings for the expert group can be interpreted as consistent with the CAH if the focus target (handles) directed attention toward movement control and thereby disrupted normally automated processes (similar to what has been shown for internal foci).

For the novice group, performance was degraded compared to baseline only by the LBEX condition and the effect was temporary, disappearing by the second trial. In contrast to expert performers, novices presumably had not yet automated the whole-body coordination to match the turning of the rope to lower-body movements. It is possible that directly focusing on control of specific components of action, whether through an internal focus on the wrists or feet, or an external focus on the handles, was necessary for success. These results were consistent with previous research. For example, Castaneda and Gray (2007) showed that less skilled batters performed most accurately using cues that directed their attention toward certain aspects of the batting movement, regardless of whether the focus was directed internally or externally. The two upper-body focus conditions facilitated performance for the novices compared to the respective lower-body focus conditions. Specifically, the UPIN condition enhanced performance compared to the LBIN condition while the UPEX condition enhanced performance compared to the LBEX condition. Presumably, the proximity of the wrists and handles to the point of rope control helped guide the successful coordination of the upper- and lower-body actions. It is plausible that the lower-body stepping action was similar enough to biologically determined gait patterns that it required little to no attention. From this perspective, it would also be expected that directing attention to the feet would hurt novice performance in two ways. First, it would take attention away from controlling the rope. Second, it would disrupt automated control of the stepping action. Novice performance was also degraded when attention was directed toward the sounds made by the feet (LBEX), which suggests that focusing on controlling the rope was the most important target of attention for the novices. Speed jump roping involves a unique and demanding pattern of whole-body coordination that is unlikely to be automated without extensive practice. The lack of experience with this movement would mean that directing attention externally to an outcome should degrade performance because there are no automated control processes in place to substitute for conscious control.

The general effects of attentional focus instructions on performance by experts and novices remains an unresolved issue. The current results indicated that the attentional focus cues either had no effects or degraded performance for the experts compared to their normally adopted attentional strategies. Results also suggested that participants who are in the earliest learning stages may benefit from instructions which direct attention toward control of one of the limiting aspects for complex yet-to-be-automated movement skills. Further research is needed, however, to establish if this advantage can emerge compared to baseline performance.

Questionnaire results indicated that perceived adherence was high in all conditions. Responses regarding preference and perceived helpfulness were closely linked to performance for both experts and novices. Experts cited the LBEX condition (LBEX) most frequently as preferred and helpful while novices cited the UPIN and UPEX conditions most frequently as preferred and helpful. Future research should be directed toward better understanding how attentional focus instructions influence performers of different skill levels. One possible direction is to determine the focus strategies normally used by expert jump rope athletes during single-rope speed jumping so that contrasts between self-selected and instructed attentional focus conditions can be identified. Such contrasts will presumably provide insight into how instructed conditions might be disruptive to attention and performance. Additionally, it would also allow researchers to work with athletes to develop effective focus cues to enhance performance beyond self-selected approaches.

As with all attentional focus research, the current study had a number of limitations. A variety of different counterbalancing strategies have been adopted in previous work. Counterbalancing has typically only been acknowledged as a means of distributing potential order effects. Although this is an important consideration, it is not the only concern. The current study adopted a counterbalancing strategy designed to also distribute potential fatigue effects. In doing so, all four conditions were presented once and then the sequence was repeated. Logically, this strategy may have facilitated comparisons of cues by participants who could have covertly switched cues. Given that there is no objective way to validate adherence, the potential for covert cue abandonment is an issue in all attentional focus research. Participants in the current study reported relatively high levels of perceived success in using the cues. Because the questionnaire asked for comparisons, it was administered after all trials were completed. It is unknown whether this procedure produced different results than if participants reported perceived success immediately following each trial. Although there is no way to determine if self-reported adherence is an accurate reflection of actual behavior, it is reasonable to suspect that responses to the questionnaire may have been influenced by both the delay and intervening conditions. Future work will be needed to determine if immediate self-reports differ from end-of-study reports. Another important issue in attentional focus research relates to the development of cues. Carson et al. (2016) and Collins et al. (2016) have expressed concern that attentional focus research does not represent demands seen in practical performance settings. In the current study, cues were intentionally not matched for experts and novices in recognition of their different attentional needs. Cues were developed in consultation with competitive athletes and coaches who instruct all skill levels, and differences were consistent with the needs of different stages of learning (Fitts and Posner, 1967). The direct comparison of experts and novices was not the primary focus of the current study and so it was deemed appropriate to develop cues that generally aligned with the distinction between internal and external foci. For other research questions, cue matching may be theoretically important. More work is needed to fully understand how the properties of specific cues might impact attentional focus effects. The familiarity findings in the current study illustrated that specificity was important. Although the cues were consistent with the target areas commonly used in the discipline, many of the experts reported that the specific cues were novel to them.

The current study produced a number of results that were consistent with previous research and others that diverged from previous findings. The deleterious findings for experts compared to baseline and the lack of differences between conditions was consistent with other research using experts (Wulf, 2008; Porter and Sims, 2013; Winkelman et al., 2017). The lack of an external focus benefit for the novices was not consistent with the majority of previous research on attentional focus effects (Wulf, 2013). A novel result from this study was that the upper-body conditions facilitated performance compared to the lower-body conditions for novices, regardless of focus direction (internal or external). Caution is warranted in terms of practical application, because none of the conditions produced lasting changes compared to baseline. Nevertheless, the pattern of results suggests that the task of speed jump roping may present some particular control challenges that do not align well with the distinction between internal and external. Accordingly, it may serve as a useful task in further examinations of how internal and external cues can prompt conscious control and whether or not such a control strategy might be helpful when first learning some types of tasks.

## Ethics Statement

This study was carried out in accordance with the recommendations of the University of Tennessee Institutional Review Board with written informed consent from all subjects. All subjects gave written consent in accordance with the Declaration of Helsinki. The protocol was approved by the University of Tennessee Institutional Review Board.

## Author Contributions

JF and KC designed the study, analyzed data, and wrote the manuscript. KC collected all the data.

## Conflict of Interest Statement

The authors declare that the research was conducted in the absence of any commercial or financial relationships that could be construed as a potential conflict of interest.

## Footnotes

1 The current study examined focus cues that were consistent in terms of general target areas (i.e., upper-body internal, upper-body external, lower-body internal, and lower-body external) but not matched exactly in terms of cue wording for the experts and novices.

2 A single baseline trial was used to minimize the potential effects of fatigue on performance. A second baseline trial would have increased exercise time by over 11%.

3 The counterbalancing procedure was used to distribute any potential order effects inherent in within-subjects attentional focus study designs. The baseline trial was not included in the counterbalancing so as to preclude possible use of a previously presented experimental cue (cf. McNevin and Wulf, 2002; Wulf et al., 2007).

4 Cues were developed based on pilot testing and in consultation with expert athletes and coaches who work with all skill levels. The intent was to provide cues consistent with how attention is commonly directed in practical settings (e.g., to the wrists, handles, feet, or sounds) and which also aligned with the categorical distinction between internal and external.

5 Two different experts experienced a broken rope during a single trial, one during UPIN-1 and one during LBIN-2. Missing data for these trials were replaced with the median value for the group during the respective trials.
